# Organoid co‐culture models of the tumor microenvironment promote precision medicine

**DOI:** 10.1002/cai2.101

**Published:** 2023-12-17

**Authors:** Zhaoru Gu, Quanyou Wu, Bingqing Shang, Kaitai Zhang, Wen Zhang

**Affiliations:** ^1^ State Key Laboratory of Molecular Oncology, Department of Etiology and Carcinogenesis, National Cancer Center, National Clinical Research Center for Cancer, Cancer Hospital Chinese Academy of Medical Sciences and Peking Union Medical College Beijing China; ^2^ Department of Urology, National Cancer Center, National Clinical Research Center for Cancer, Cancer Hospital Chinese Academy of Medical Sciences and Peking Union Medical College Beijing China; ^3^ Department of Immunology, National Cancer Center, National Clinical Research Center for Cancer, Cancer Hospital Chinese Academy of Medical Sciences and Peking Union Medical College Beijing China

**Keywords:** air‐liquid interface, immunotherapy, organoid, tumor microenvironment, tumor‐on‐chip

## Abstract

In recent years, the three‐dimensional (3D) culture system has emerged as a promising preclinical model for tumor research owing to its ability to replicate the tissue structure and molecular characteristics of solid tumors in vivo. This system offers several advantages, including high throughput, efficiency, and retention of tumor heterogeneity. Traditional Matrigel‐submerged organoid cultures primarily support the long‐term proliferation of epithelial cells. One solution for the exploration of the tumor microenvironment is a reconstitution approach involving the introduction of exogenous cell types, either in dual, triple or even multiple combinations. Another solution is a holistic approach including patient‐derived tumor fragments, air‐liquid interface, suspension 3D culture, and microfluidic tumor‐on‐chip models. Organoid co‐culture models have also gained popularity for studying the tumor microenvironment, evaluating tumor immunotherapy, identifying predictive biomarkers, screening for effective drugs, and modeling infections. By leveraging these 3D culture systems, it is hoped to advance the clinical application of therapeutic approaches and improve patient outcomes.

## Graphical abstract

Three dimensional (3D) culture is a promising model for tumor research, replicating solid tumor characteristics. Advantages include high throughput and retention of heterogeneity. Traditional matrigel‐submerged organoid culture requires exogenous cells to study the tumor microenvironment (TME). A holistic approach using patient‐derived tumor fragments (PDTFs), air‐liquid interface (ALI), suspension 3D culture, and microfluidic tumor‐on‐chip (ToC) models better mimic the tumor structure and composition. Organoid co‐culture models are useful for studying the TME, immunotherapy, biomarkers, drug screening, and infections. These 3D systems aim to improve therapeutic approaches and patient outcomes.

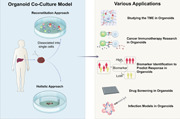

**Abbreviations**
ATO: artificial thymic organoid
BME: basement membrane extract
CAFs: cancer‐associated fibroblasts
CAR: chimeric antigen receptor
CCA: cholangiocarcinoma
CRC: colorectal cancer
CTLs: cytotoxic T lymphocytes
DCs: dendritic cells
DKK1: Dickkopf 1
ECM: extracellular matrix
EMT: epithelial‐mesenchymal transition
GBM‐BCO: glioblastoma‐brain cortical organoid
GBOs: glioblastoma organoids
GSC: glioblastoma stem cell
HANS: high‐affinity neoantigens
HCC: hepatocellular carcinoma
HER: human epidermal growth factor receptor
ICIs: immune checkpoint inhibitors
IL: interleukin
iPSCs: induced pluripotent stem cells
ITM: immunosuppressive tumor microenvironment
MDSCs: myeloid‐derived suppressor cells
MEK‐I: MEK inhibitors
MHC: major histocompatibility complex
NK: natural killer
NRG1: neuregulin 1
NSCLC: nonsmall cell lung cancer
OoC: organ‐on‐chip
OS: overall survival
PBLs: peripheral blood lymphocytes
PBMCs: peripheral blood mononuclear cells
PDAC: pancreatic ductal adenocarcinoma
PDOs: patient‐derived organoids
PDTOs: patient‐derived tumor organoids
PDTXs: patient‐derived tumor xenografts
PD‐1: programmed cell death protein 1
PD‐L1: programmed cell death ligand 1
PMN‐MDSC: polymorphonuclear‐myeloid‐derived suppressor cell
PSC: pluripotent stem cell
TAMs: tumor‐associated macrophages
TCRs: T‐cell receptors
TGFβ: transforming growth factor‐beta
TILs: tumor‐infiltrating lymphocytes
TLS: tertiary lymphoid structures
TME: tumor microenvironment
TNF: tumor necrosis factor
ToC: tumor‐on‐chip
ZIKV: Zika virus
αSMA: alpha‐smooth muscle actin

## 1 INTRODUCTION

Recurrence and metastasis are responsible for approximately 90% of cancer‐related deaths [1, 2, 3, 4]. However, improvements in therapy have not kept pace with disease progression, making cancer a major focus of biomedical research [5]. Cancer cell lines have certain limitations, including the accumulation of multiple mutations during culture [6], failing to establish permanent cell lines from solid tumors, inability to mimic the complexity of the tumor microenvironment (TME), and showing multiple resistance mechanisms in monolayer cultures [7]. Patient‐derived tumor xenografts also have drawbacks, including being costly and time‐consuming, lacking immune cell regulation in tumors, causing selective engraftment of tumors [7], and having variable engraftment efficiency across tumors, which hinder their use for high‐throughput screening [6]. Therefore, alternative models that capture the complexity of the TME are needed to advance preclinical research on related therapies. This has significant implications for finding new treatments, identifying individuals who respond to immunotherapy, and stratifying patients [8].

**Figure 1:**
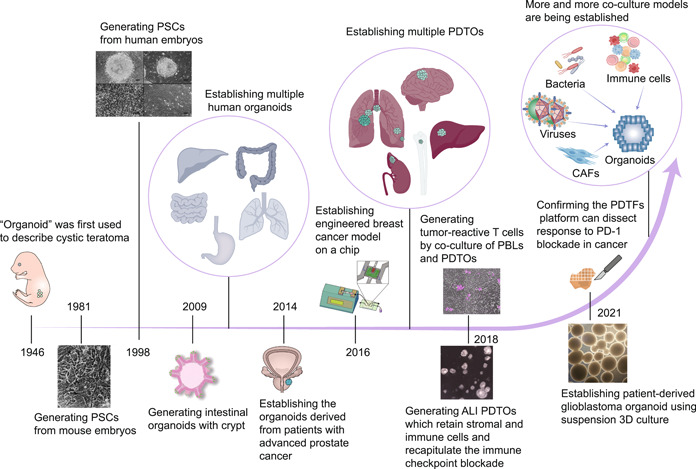
History of the development of organoid and co‐culture systems. The history of organoids and cancer organoids and the well‐established co‐culture systems are shown for various cancer organoids and specific cell types. Images adapted with permission from references [20, 21, 22, 23], Copyright Cell Press. Images adapted with permission from [24, 25], Copyright Nature Publishing Group. Images adapted with permission from [26], Copyright American Association for the Advancement of Science. Copyright Nature Publishing Group. Images adapted with permission from [27], Copyright John Wiley and Sons Ltd.

The term “organoid” was coined as far back as 1946 by Smith and Cochrane to describe cystic teratoma [9] (Figure 1). Recent studies have characterized “organoids” as self‐organized three‐dimensional (3D) structures derived from stem cells that contain multiple cell types specific to organs, mimic the spatial organization of organs, and can recapitulate some of their functional aspects [10, 11]. Gene profiling and histopathological analysis have shown that patient‐derived tumor organoids (PDTOs) preserve the genetic diversity and phenotypic heterogeneity of the original tumor more faithfully than cancer cell line models. Moreover, PDTOs are a promising alternative to patient‐derived tumor xenografts for preclinical assays because they can perform clinical analyses, such as high‐throughput drug screening, with fewer resources [12]. Xenografts in immunocompromised mice are widely used for assessment of the toxicity of cancer chemotherapy but their clinical relevance is limited, resulting in the failure of numerous trials as a result of efficacy and safety concerns [13, 14]. Organoid technology has emerged as a promising model for evaluating chemotherapy‐induced toxicity within a patient‐specific context. By identifying biomarkers of damage in traditionally implicated organs like the liver, kidney, and vascular system, organoids provide a valuable way of overcoming these challenges and enhance translational research [15, 16, 17, 18]. Hence, these advantages make them a more reliable in vitro model for simulating human cancer, predicting in vivo drug sensitivity, and monitoring tumor progression [19, 20].

However, a traditional Matrigel‐submerged culture can only achieve long‐term expansion of epithelial‐derived tumors. Therefore, co‐culture models are needed to better mimic the real TME [28, 29]. This review focuses on organoid co‐culture techniques used for various purposes, such as studying cell–cell interactions in the TME, exploring cancer immunotherapy, identifying biomarkers that can predict a patient's response, drug screening, and using preinfection models as a preclinical reference for cancer research.

## 2 WHAT IS AN ORGANOID CO‐CULTURE MODEL?

### 2.1 Reconstitution approach

**Figure 2:**
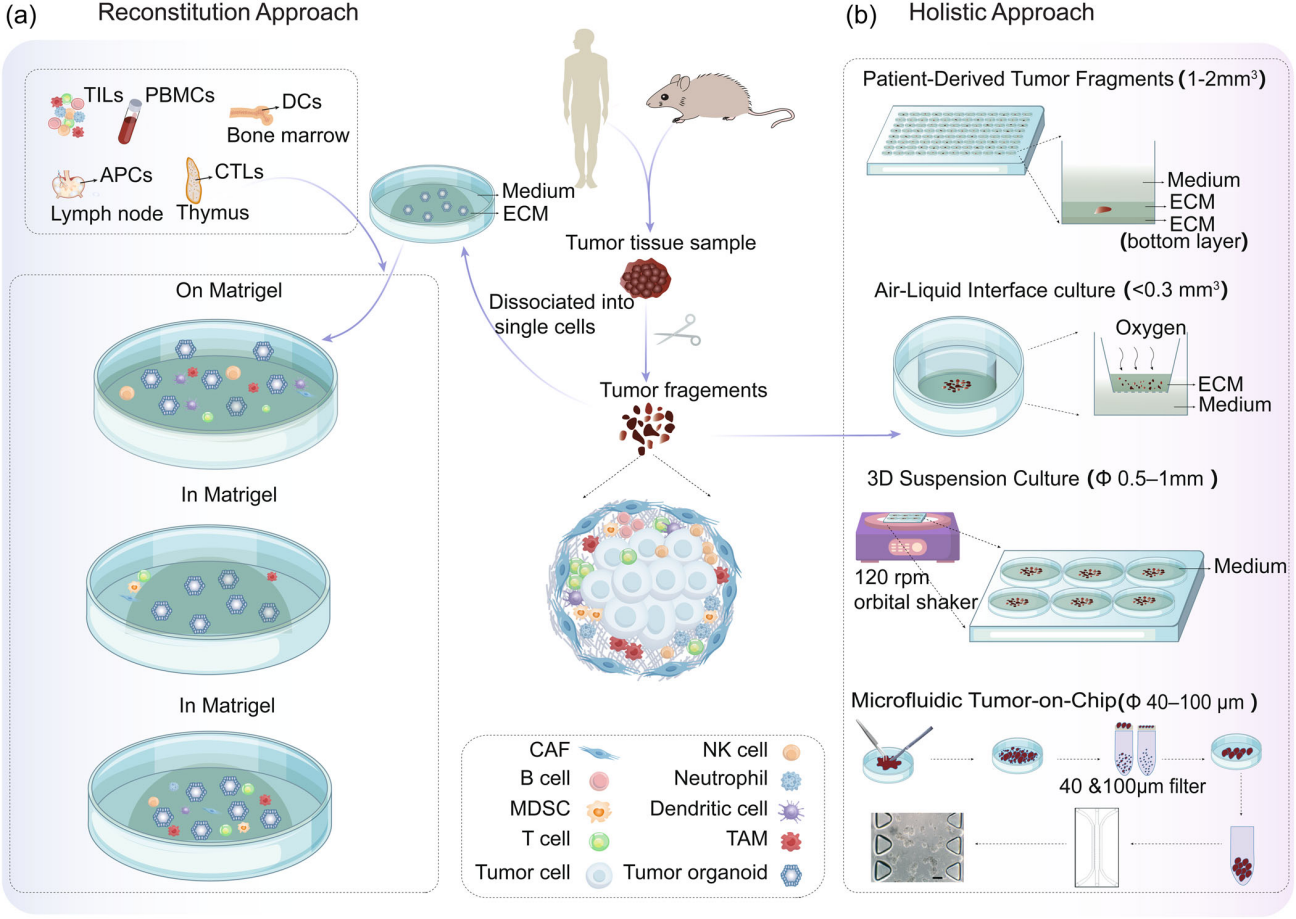
Different organoid co‐culture models are used to simulate the tumor microenvironment. (a) In the reconstituted method, epithelial‐derived tumor cells in tissues are mechanically or enzymatically dissociated and expanded by immersion culture to form PDTOs. TILs, PBMCs, DCs, antigen‐presenting cells, CTLs, and other cells of interest are isolated, expanded, cultured, and then co‐cultured with PDTOs according to the purpose of the research. The main method is to submerge PDTOs in the ECM, submerge the cells of interest in the medium for indirect co‐culture or immerse them in the ECM for direct co‐culture, or suspend PDTOs and cells on the ECM. (b) The holistic approach uses cancer organoids cultured directly from tumor fragments while preserving endogenous immune cells and other nonepithelial cell types. In the PDTF platform, 1–2 mm^3^ of tumor fragments are cut from different regions within the tumor, and individual PDTFs are embedded in artificial ECM, after which the medium is added on top. ALI culture grows mechanically dissociated tissue (<0.3 mm^3^) on top of the ECM inserted inside the culture well, and the medium is provided from the outer culture well through a permeable membrane. The top of the insert is in direct contact with the air. Suspension three dimensional (3D) culture grows tumor fragments (0.5–1 mm in diameter) in the ECM‐free medium and is cultured on a 120 rpm orbital shaker at 37°C and in 5% CO_2_. The microfluidic ToC platform is generated by co‐culturing tumors and stromal cells or tumor fragments (40–100 μm in diameter) in a chamber that can be continuously perfused in a 3D biomimetic matrix in a microfluidic device. ALI, air‐liquid interface; CTLs, cytotoxic T lymphocytes; DCs, dendritic cells; ECM, extracellular matrix; PBMCs, peripheral blood mononuclear cells; PDTFs, patient‐derived tumor fragments; PDTOs, patient‐derived tumor organoids; TILs, tumor‐infiltrating lymphocytes; ToC, tumor‐on‐chip.

Various co‐culture methods have been developed for specific purposes (Figure 2a), including investigating intercellular interactions [30], immunotherapy [31], and patient‐specific cytotoxicity [32]. The primary method involves isolating and expanding epithelial tumor cells from tissues and co‐culturing them with cells such as cancer‐associated fibroblasts (CAFs), tumor‐infiltrating lymphocytes (TILs), or peripheral blood mononuclear cells (PBMCs), either in dual, triple or even multiple combinations.

#### 2.1.1 Dual co‐culture models

First, dual co‐culture models allow for the investigation of specific cell–cell interactions. For example, PDTOs can assess toxicity when co‐cultured with T‐cells. One study co‐cultured rectal cancer organoids and TILs and found that cytotoxicity was enhanced to a greater extent when TILs were embedded in the matrix than when they were added to the medium [32]. Another study reported a method for expanding and characterizing tumor‐reactive T‐cells using organoid culture [21, 33]. They generated tumor organoids, exposed them to interferon‐gamma (IFN‐γ) for 24 h, and then co‐cultured them with autologous peripheral blood lymphocytes. This study demonstrated the feasibility of continuous production of patient‐derived T‐cell products and, surprisingly, that these tumor‐specific T‐cells recognized and eliminated autologous tumor organoids but not normal ones [21, 33], indicating great promise for future immunotherapies.

Furthermore, many researchers have explored and optimized co‐culture methods and medium components for investigation of the killing of tumor organoids by natural killer (NK) cells [31] and T‐cells [34]. For example, colon cancer organoids were embedded in Matrigel, cultured on a thin layer of Matrigel, or submerged in suspension before adding NK cells to investigate the best co‐culture method. Microscopic examination revealed that effector cells migrated on the surface of the Matrigel but could not penetrate the dense extracellular matrix (ECM). After 8 h, culture on thin Matrigel increased migration of NK cells and stabilized effector‐target cell interactions when compared with culture in suspension, leading to significant chimeric antigen receptor (CAR)‐induced lysis [31]. This was consistent with the poor infiltration capacity of NK cells in vivo [35], which may have been attributable to the high levels of ECM and fibrous tissue surrounding the tumor. In contrast, T‐cells added to the medium surrounding cholangiocarcinoma (CCA) organoids immersed in the basement membrane extract (BME) dome for 7 days destroyed the organoids without direct contact [34]. This killing may be mediated by soluble factors produced by T‐cells with patient‐specific specificity, revealing that T‐cells can act as a killer independent of ECM, unlike NK cells [31]. As with NK cells, co‐culture in a 10% BME suspension preserves 3D organoid morphology and allows direct interaction of PBMCs with organoids [34]. Furthermore, the study optimized medium conditions for PDTOs and T‐cell survival and function, emphasizing that nicotinamide‐free organoid medium should be used and 10% human serum should be added to a 10% BME suspension [34]. These optimization strategies have paved the way for accelerated translational research.

#### 2.1.2 Triple and multiple co‐culture models

Researchers have also established a triple co‐culture scheme of tumor organoids with T‐cells and other immune cells to assess toxicity. Chakrabarti and colleagues [36, 37] isolated dendritic cells (DCs) and cytotoxic T lymphocytes (CTLs) from mouse bone marrow and thymus, respectively, and cultured them with tumor organoids. They found that Hedgehog signaling induced expression of programmed cell death ligand 1 (PD‐L1) and proliferation of tumor cells in gastric cancer. When pulsed DCs and CTLs were co‐cultured with organoids in the presence of an anti‐PD‐L1 antibody, immune cells migrated to the organoids, and there was significant apoptosis only in tumor organoids. The research team then applied this co‐culture model in patients [38, 39] and demonstrated that PDTO/immune cell co‐cultures may be used to study the immunosuppressive function of myeloid‐derived suppressor cells (MDSCs) within the gastric TME, providing a more comprehensive model for investigating the complex roles of other cells in the TME.

A recent study described a platform that integrates patient‐specific mature lymph node antigen‐presenting cells into organoids without cell sorting to generate adaptive immunity [40, 41, 42]. When matched peripheral blood T‐cells from patients were exposed to co‐cultures, they became activated and developed memory for various heterogeneous tumor neoantigens. These trained T‐cells efficiently destroyed tumor cells in naive tumor organoids from the same patient [42]. The researchers screened immune‐enhanced and nonenhanced PDTOs with checkpoint inhibitors and found increased destruction of immune‐enhancing organoids. When the immune‐enhanced PDTO response was compared with the patient's clinical response to immunotherapy, there was a correlation in 85% of cases [40]. This platform uses immune‐enriched tumor organoids to test the efficacy of immunotherapies and represents a major advance in patient‐specific immune organoid culture, providing a reliable and efficient model for the development of personalized immunotherapy [40, 41, 42].

In summary, choosing the appropriate method for immuno‐organoid co‐culture depends on downstream assays. Short‐term organoid immune cell co‐culture is appropriate for therapies that function within hours. Longer co‐cultures are required for therapies that produce results over time to support tumor and immune cell viability, and the time to add immune components needs to be optimized. The composition of patient‐specific immune cells is complex and cannot be easily replicated. The source of exogenous immune cells can be patient‐specific PBMCs or allogeneic immune cells. Donor T‐cells that recognize major histocompatibility complex (MHC) molecules on organoid cells as nonself pose a challenge in allogeneic cultures, resulting in high background killing compared with autologous systems and impairing assay specificity. Autologous co‐culture or human leukocyte antigen (HLA)‐matched immune cells are required if T‐cell antigen presentation is to be detected.

### 2.2 Holistic approach

The holistic approach, including patient‐derived tumor fragments (PDTFs), air‐liquid interface (ALI) culture, suspension 3D culture, and microfluidic tumor‐on‐chip (ToC), contains more complex endogenous stromal and immune compartments, which are needed to model the native immune TME and assess the efficacy of immunotherapy [43] (Figure 2b).

#### 2.2.1 Patient‐derived tumor fragments

The PDTF platform characterizes early antiprogrammed cell death protein 1 (PD‐1)‐induced immune changes in tumors by embedding small tumor fragments in a collagen and Matrigel ECM to prevent efflux of lymphocytes and treating them ex vivo with anti‐PD‐1 for 48 h [44, 45]. The treated fragments are then assayed for induction of cytokines, chemokines, and markers of T‐cell activation, allowing for creation of an unbiased immunological score that distinguishes PDTF responders from nonresponders. This score can be retrospectively correlated with clinical outcomes in patients who receive anti‐PD‐1 treatment. A recent report showed 100% concordance between ex vivo PDTF immune responses and clinical outcomes [22]. This method has been used to create PDTFs from various types of cancer, including melanoma, nonsmall cell lung cancer, and breast, ovarian, and renal cell carcinoma. However, this method has some caveats, including the absence of radiographic confirmation in some cases and the possibility of heterogeneous PDTF immune responses by different tumor lesions in the same patient [22].

While PDTFs hold promise for investigating tumor biology and treatment responses, it is important to acknowledge their inherent limitations. First, challenges arise in acquiring and processing samples, with factors such as tumor location, size, and morphology potentially complicating the collection and handling of patient tumor tissue samples, thereby affecting the establishment of PDTFs. Second, there is concern regarding cell heterogeneity, because PDTFs may not fully capture the diverse nature of the patient's tumor tissue owing to possible loss of specific tumor subsets during the culture process. In conclusion, although PDTFs have advantages, researchers must carefully consider these limitations. Thoughtful experimental design and data interpretation are essential when utilizing such models, which should align with the objectives of the study.

#### 2.2.2 Air‐liquid interface culture

To fully harness the potential of organoids in tumor immune research, it is essential to investigate the feasibility of constructing an immune TME within organoids. Finnberg et al. [46] developed ALI cultures from surgical tumor specimens obtained from patients with colorectal cancer (CRC) or lung cancer. ALI culture involves mechanically dissociated tissue placed atop a type I collagen matrix within a culture well. Medium is introduced from an adjacent well through a permeable membrane, and the upper surface of the matrix interacts directly with air, facilitating diffusion of oxygen [47, 48, 49]; this likely contributes to the ability of ALI cultures to grow substantial multicellular organoids while preserving their inherent tissue architecture. Stromal cells inherently sustain organoid growth within ALI culture, eliminating the need for supplementary growth factors through the production of essential endogenous agents [47]. Finnberg et al. [46] integrated peripheral and tumor‐derived immune cells into the in vitro tumor cultures to assess their ability to mimic the immunosuppressive TME. While CD45^+^ cells were readily detectable in 3D ALI cultures, a significant reduction in CD3^+^ cells was observed. Moreover, only growth of CAFs was recorded in squamous 3D ALI nonsmall cell lung cancer cultures. This is certainly an interesting development in the field of tumor immune research.

A subsequent report further optimized ALI culture [23]. ALI PDTOs can grow from various tumor sites and preserve the tumor epithelium and its matrix microenvironment in vitro for at least 30 days [23, 50]. Notably, a decrease in interstitial myofibroblasts was observed in 70% of renal and colon tumor cultures. Immune components of cells were maintained for up to 30 days in organoid cultures. The heterogeneity of T‐cell receptors (TCRs) found in primary tumors was also preserved. The authors used these organoids to model immune checkpoint blockade, leading to the proliferation and activation of tumor antigen‐specific T‐cells and subsequent tumor killing. PDTOs offer opportunities for in vitro modeling of human immunotherapy [23]. In contrast with the tumor epithelium that can be continuously passaged and cryopreserved, the immune component of ALI PDTOs declines over time, which does not last longer than approximately 2 months despite supplementation with interleukin (IL)‐2 [23].

Although the ALI model has successfully retained stromal and immune cells for at least a month in various tumors, questions remain about its transformability. Researchers have used surgically dissected samples to isolate the test population without evaluating significant parallel patient treatment controls since researchers have not been comparing their experimental setups with equivalent conditions observed in actual patients undergoing treatments. This leaves gaps in the expected correlation between simulating the TME and patient immunotherapy responses. Furthermore, ALI techniques cannot address the limitation that immune and stromal components in organoid cultures can only be maintained for a short time; this is partly because of the rapid reduction of the stromal cell bank, making long‐term studies of epithelial cell‐immune cell interactions difficult.

#### 2.2.3 3D suspension culture

A recent study showed that fragments of glioblastoma from patients can be expanded into organoids using a serum, epidermal growth factor/basic fibroblast growth factor, and ECM‐free medium on a 120 rpm orbital oscillator at 37°C and 5% CO_2_ [20]. These patient‐derived glioblastoma organoids accurately reproduce the histological features, cellular heterogeneity, gene expression patterns, and mutation profiles of their original tumors. This culture method enables the rapid and reliable generation and biobanking of glioblastoma organoids, providing an abundant resource for both basic and translational glioblastoma research [51]. Nevertheless, this suspension culture may not faithfully replicate the nutrient, oxygen, and other gradients seen in solid tumors. Furthermore, it frequently lacks the mechanical forces and physical cues inherent in solid tumors, which are factors that can profoundly impact tumor growth, behavior, and drug response. These discrepancies could potentially result in deviations from in vivo conditions.

#### 2.2.4 Microfluidic tumor‐on‐chip

A limitation of organoid cultures is their lack of reproducibility owing to variations in size, shape, cell count, and geometry. To address this issue, emerging organ‐on‐chip technologies, particularly ToC, have been developed by combining cell biology with advanced techniques like micromachining and microfluidics, which provide a stable and reproducible platform, to improve consistency and control for enhanced use in high‐throughput screening and testing [52]. The ToC platform is created by co‐culturing tumor and stromal cells in a continuously perfused chamber within a 3D biomimetic matrix on a microfluidic device [53]. ECM‐mimicking culture scaffolds provide structural and functional support to promote cell survival, proliferation, and differentiation [54], allowing ToC models to recapitulate key mechanobiological features of the TME for studying the collective migration and invasion of cancer cells [55]. The microfluidic device can also generate chemokine gradients for studying the chemotaxis of immune cells toward the tumor nest and distal metastasis of tumor cells [53, 56].

The combination of ToC technology, which allows PDTOs to exhibit diverse functionalities and structures corresponding to individual patients and types of cancer, along with the advantages of microfluidic technology, enables precise monitoring and control, enhancing the relevance of cancer immunotherapy screening [57]. For example, Jenkins et al. [58] have described a 3D microfluidic device for the short‐term culture of mouse‐derived and patient‐derived organotypic tumor spheroids with preserved immune cell populations. The sensing device allows for real‐time monitoring of tumor‐immune interactions, delivery of checkpoint inhibitors and small‐molecule drugs through microfluidic channels, and live/dead screening and cytokine analysis. Their study demonstrated ex vivo sensitivity and resistance to PD‐1 blocking therapy within a short period (3–6 days), highlighting the potential for rapid evaluation of immune checkpoint blockade‐based therapies in a clinical setting [58]. Another study used a microfluidic system to screen small molecules that enhance T‐cell activity under inhibition of PD‐1 [59]. Treatment with a CDK4/6 inhibitor increased infiltrating T‐cell levels and synergized with anti‐PD‐1 blocking antibodies to enhance antitumor activity [59]. Moreover, by establishing an in situ breast tumor model with varying levels of PD‐L1 expression, it was shown that on‐chip interrogation of primary tumor responses to PD‐1 as early as 10 days post‐tumor inoculation could predict in vivo tumor responses to PD‐1 at Day 24 [60].

In addition to recapitulating the TME, microfluidic devices can encapsulate an important aspect of the immune environment, namely, vascularization. Organoids in culture reach a finite size beyond which oxygen, nutrient, and metabolite exchange can no longer rely solely on diffusion. This leads to the formation of a central hypoxic core [61, 62]. Hypoxic cores can be observed in organoids or spheres larger than 100–200 μm in size, and necrosis occurs in organoids larger than 500 μm in diameter [63]. Therefore, achieving the vascularization of organoids remains a major challenge in terms of maintaining their complexity and scale. Vascular co‐culture can be added in vitro through layer‐by‐layer deposition of endothelial cells or by selectively removing material to form tubular voids seeded with endothelial cells connected to the perfusion network. More complex organoids containing vascular‐like structures can also be created [54, 64]. Alternatively, angiogenesis can be induced and guided by hypoxia gradients in vascular endothelial growth factor using a separate microfluidic chip for organoid‐endothelial cell co‐cultures [54]. Organoid and vascular co‐cultures have been extensively investigated across various cancer types in academic research. For example, a microfluidic model of breast cancer has been developed to assess the effect of TME on the response to treatment and extravasation of cancer cells in breast cancer [65]. Mannino et al. [66] created a microfluidic model of diffuse large B‐cell lymphoma with endothelialized blood vessels and separate lymph node lumens to mimic tumor‐immune interactions as well as the vasculature. Roy's laboratory has also developed an on‐chip lung cancer model containing a microvasculature and fibroblasts [67].

In summary, ToC models enhance the reproducibility of studies and recapitulate key mechanobiological features in the TME, including the vasculature, thereby improving the relevance of cancer immunotherapy screening. Nevertheless, ToC has limitations that require recognition and consideration. These include high technical complexity, significant costs, challenges in establishing large‐scale tumor models owing to limited dimensions, lack of standardized protocols leading to poor reproducibility and comparability, and difficulties with long‐term cultivation, which restrict comprehensive investigation of prolonged effects and dynamic changes.

## 3 SELECTION OF THE ORGANOID CO‐CULTURE METHOD ACCORDING TO PURPOSE

### 3.1 Studying the TME in organoids

**Figure 3:**
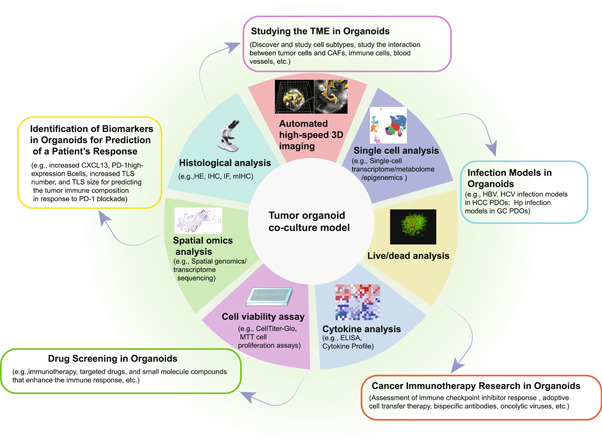
Downstream applications of organoid co‐culture models. Direct or indirect co‐culture of PDTOs with cells of interest, as well as holistic co‐culture platforms (e.g., PDTO, ALI, 3D suspension culture, and ToC), can be used to study the TME using a variety of analytical methods, evaluate immunotherapy studies, identify biomarkers, and establish infection models to research‐related tumorigenic factors. Images adapted with permission from [69], Copyright Nature Publishing Group. ALI, air‐liquid interface; PDTOs, patient‐derived tumor organoids; TILs, tumor‐infiltrating lymphocytes; TME, tumor microenvironment; ToC, tumor‐on‐chip.

Complex interactions between CAFs, immune cells, blood vessels, and tumor cells promote the production of different subtypes of CAF and drive tumor progression, metastasis, drug resistance, and treatment failure in most solid tumors. Organoids have become a suitable platform for studying these interactions [68] (Figure 3).

Tumor cells promote production of multiple subtypes of CAF through different mechanisms. Öhlund et al. [70] co‐cultured patient‐derived pancreatic ductal adenocarcinoma (PDAC) organoids with pancreatic stellate cells and found that the organoids could promote their differentiation into two CAF subsets. Subpopulations close to tumor cells strongly express alpha‐smooth muscle actin (αSMA) while those far from tumor cells strongly express IL‐6 and other inflammatory mediators. Further studies showed that organoid‐secreted transforming growth factor‐beta and downregulation of IL1R1 led to differentiation of stellate cells into αSMA^+^ CAFs, while organoid‐secreted IL‐1‐induced expression of leukemia inhibitory factor and the activated JAK/STAT signaling pathway enabled differentiation into inflammatory CAFs [71]. Another study identified new subsets of CAF directly involved in immune processes functioning as antigen‐presenting CAFs [30].

In addition to the impact of tumor cells on CAFs, it is noteworthy that CAFs have diverse effects on tumor cells and can potentially promote tumor resistance. Researchers found that CAFs could maintain the proliferation of CRC patient‐derived organoids (PDOs) and restore biological pathways present in patient tissue but absent in individual PDO cultures without adding growth factors [72]. Likewise, Seino et al. [73] found that Wnt secreted by CAFs could promote the growth of PDAC PDO subtypes unable to autocrine Wnt, while Wnt‐secreting PDAC PDO subtypes autonomously create their own Wnt niche. Remarkably, Zhang et al. [74] co‐cultured advanced prostate cancer PDOs with CAFs and found that neuregulin 1 secreted from CAFs could activate human epidermal growth factor receptor 3 in tumor cells, resulting in resistance to second‐generation antiandrogen therapy. The findings of another study support the key role of CAF‐driven induction of epithelial‐mesenchymal transition in chemoresistant PDAC [75]. Indeed, the presence of CAFs affects not only tumor cells but also other cells in the microenvironment. For example, Kuen et al. [76] observed that tumor/fibroblast spheroids induced an M2‐polarized phenotype of monocytes similar to tumor‐associated macrophages in PDAC in a 3D co‐culture model. Interestingly, the spatial configuration of cells in the TME also affects cancer and fibroblast phenotypes, leading to clinical challenges concerning tumor heterogeneity and resistance to treatment [77].

In addition to CAFs, immune cells also play a significant role and can be studied in the TME by co‐culture. For example, DeNardo et al. [78] found that CD4^+^ T lymphocytes expressing IL‐4 modulated the tumor‐associated macrophage phenotype and effector function, indirectly promoting invasion and metastasis of breast cancer. Tsai et al. [79] subsequently constructed a complex organoid TME model containing tumor, stromal, and immune components and observed activation of myofibroblast‐like CAFs and tumor‐dependent infiltration of lymphocytes. Moreover, Courau et al. [80] analyzed CRC PDOs and autologous TILs to assess the infiltration, activation, and function of T‐cells and NK cells in human colorectal tumors in vitro. Introduction of pathogens or commensal microbiota into tumor organoids with immune cells enables assessment of immunomodulatory outcomes and responses to immunotherapy by recapitulating cancer‐related inflammation and carcinogenesis through pathogen‐epithelial‐immune cell interactions [81, 82].

Furthermore, researchers have investigated the relationship between tumors and blood vessels. Silvestri et al. [83] developed a tissue engineering model that incorporated microvasculature co‐cultured with mammary tumor organoids. Imaging revealed tumor organoids that were integrated into the endothelial cell lining, resulting in blood vessels with gaps in their basement membrane. Tumor organoids actively contracted and displaced blood vessels, and clusters of cancer cells underwent endocytosis following formation of mosaic vessels. These findings suggest that cancer cells can rapidly remodel, destroy, or integrate into existing blood vessels, impacting oxygenation, perfusion, and systemic transmission [83]. A recent study established an organoid co‐culture model mimicking vascular secretion crosstalk between hepatocellular carcinoma (HCC) cells and endothelial cells using well‐defined hydrogel systems [84]. Endothelial cells induced HCC cells to create an inflammatory microenvironment by recruiting immune cells. This model can serve as a tool for understanding and targeting the interaction between angiogenesis and the immune environment.

### 3.2 Cancer immunotherapy research in organoids

Cancer immunotherapy involves manipulating the immune system to recognize and attack cancer cells. This can be achieved by various methods, including checkpoint inhibitors, adoptive cell therapy, bispecific antibodies, cytokines, and oncolytic viruses [85, 86]. However, at present, the effectiveness of immunotherapy in an individual patient cannot be accurately predicted until treatment is administered. For example, PD‐1 or PD‐L1 expression does not correlate reliably with the response to treatment [87], and the tumor mutation burden and loss of mismatch repair machinery indicate increased expression of tumor neoantigens [88, 89]. As a result, there is a high demand for preclinical models [53] (Figure 3).

#### 3.2.1 Immune checkpoint inhibitor response in organoids

Checkpoint inhibitors are a well‐studied class of immunotherapy [86]. These agents block proteins on the surface of cancer cells, helping them to evade the immune system, and have been widely investigated in PDTOs. For example, Scognamiglio et al. [90] found that PD‐1/CD8‐positive lymphocytes from PD‐L1‐positive organoids show promise as tools for immune checkpoint inhibition. However, considering that immune checkpoint inhibitors (ICIs) are not always effective, researchers have developed combination therapies to overcome immune resistance. These approaches involve targeting specific molecules within pathways and directly modulating immune cells to induce antitumor effects. Researchers have attempted to enhance the efficacy of ICIs by inhibiting pathways targeting specific molecules. For example, Della Corte et al. [91] confirmed that MEK inhibitors regulate the immune microenvironment by downregulating expression of PD‐L1, enhancing expression of MHC‐I on tumor cells, and increasing the production of several cytokines, including IFN‐γ, IL‐6, IL‐1β, and tumor necrosis factor‐alpha, which recruit immune cells to the tumor site, triggering a broader antitumor immune response. Another approach involves targeting Dickkopf 1 (DKK1), which is associated with tumor progression [92]. Sui et al. [93] found that DKK1 suppresses CD8^+^ T‐cell antitumor immunity through the GSK3β/E2F1/T‐bet axis. Neutralizing DKK1 may restore sensitivity to PD‐1 blockade in DNA mismatch repair‐deficient CRC [93]. Although half of PD‐L1‐positive gastric tumors co‐express HER2, the interaction between HER2 and PD‐1/PD‐L1 in gastric cancer remains undetermined [94]. Chakrabarti et al. [38] used autologous gastric cancer organoids co‐cultured with CTLs and MDSCs to study this interaction. They found that inhibiting expression of HER2 decreased PD‐L1 levels in organoids, increased proliferation of CTLs, and enhanced killing of tumor cells, suggesting that co‐expression of HER2 and PD‐L1 may contribute to tumor immune escape [38]. Koikawa et al. [95] similarly discovered that Pin1 promoted proliferation of fibroblasts and an immunosuppressive TME when indirectly co‐cultured with PDAC PDOs. Furthermore, Pin1 induced lysosomal degradation of both PD‐L1 and the gemcitabine transporter equilibrative nucleoside transporter 1, which helps cancer cells take in the chemotherapy drug gemcitabine and activates multiple cancer pathways. Targeting Pin1 with clinically available drugs led to complete elimination or sustained remission of invasive PDAC when performed in combination with anti‐PD‐1 and gemcitabine.

Recently, researchers have started work on enhancing the response to ICIs by modulating immune cells. For example, one study performed a differential transcriptome analysis of tumors with variable potential for immune escape and co‐cultured tumor organoids with PBMCs91. Functional tests showed that organoids treated with ICIs and dexamethasone recruited more CD3^+^ and CD8^+^ T‐cells and expressed higher levels of cleaved caspase 3 and cleaved caspase 8, suggesting that T‐cells enhanced immune surveillance and highlighting the potential of dexamethasone to improve the response to ICIs [96]. Likewise, the inhibition of immunosuppression of polymorphonuclear MDSCs by the tyrosine kinase inhibitor cabozantinib sensitized gastric cancer organoids to nivolumab and mubritinib in co‐cultures [39, 97].

#### 3.2.2 Adoptive cell transfer therapy in organoids

Tumors are usually infiltrated by T lymphocytes that recognize self or mutated antigens but are usually inactive, although they often show signs of prior clonal expansion [98]. To activate TILs, Yin et al. [99] prepared nanoparticles containing innate immune stimulators that were able to induce strong activation of endogenous T‐cells in PDTOs. However, this activation has often been limited, leading to the development of immune cell therapies for adoptive cell transfer therapy, including therapeutic T‐cells, NK cells, DCs, and macrophages [100]. In adoptive cell transfer therapy, circulating lymphocytes or TILs are collected, selected or modified, expanded, and activated ex vivo before they are used in the patient [101].

PDTOs can be used to generate and expand tumor‐specific T‐cells [21, 33]. In one study, PDTOs were used to achieve antigen‐specific stimulation of T‐cells in the PBMC fraction [21]. CTLs expanded in co‐culture and efficiently destroyed PDTOs without T‐cell‐mediated cytotoxicity against healthy lung organoids generated from the same patients. This study showed that cancer organoids can produce antigens that induce proliferation and stimulation of T‐cells, and more importantly, be used to evaluate the tumor‐specific killing efficiency of T‐cells. Another study investigated circulating tumor‐targeted TCRs using autologous pancreatic tumor organoids [102]. Autologous PDTOs were stimulated with T‐cells obtained from patients' PBMCs for 2 weeks to generate organoid‐primed T‐cells. TCR sequencing revealed significant clonal expansion of T‐cells with a restricted subset of TCRs. Cloning and transferring these TCRs to heterologous T‐cells conferred tumor cell recognition and cytotoxic properties in a patient‐specific manner. This approach facilitates the creation of tumor‐specific T‐cells capable of recognizing and selectively eliminating cancer cells based on individual patient characteristics. This advance holds significant promise in the realm of cancer immunotherapy, providing a cornerstone for adoptive cell transfer therapy and the potential to enhance the efficacy of treatment for cancer at the individual level.

Organoid platforms are effective in generating CAR T‐cells. Seet et al. [103] created artificial thymic organoids that produce human‐engineered T‐cells with cancer recognition receptors and without off‐target effects, which are critical for established adoptive T‐cell therapies. This system is reproducible and scalable, potentially accelerating engineered development of T‐cell therapy [104]. Another study reported that the pluripotent stem cell (PSC) thymic organoid (ATO) system efficiently generates functional mature T‐cells from human PSCs [105]. The introduction of MHC‐I‐restricted TCRs in PSCs generates naive antigen‐specific CD8αβ^+^ T‐cells that lack endogenous TCR expression and show antitumor efficacy in vitro and in vivo. A recent study found that ATO 3D organoid cultures supported the differentiation of human CAR^+^ induced pluripotent stem cells (iPSCs) into high‐functioning CAR T‐cells [106, 107]. Expanded iPSC CD19‐CAR T‐cells, which refers to T‐cells derived from iPSCs that have been engineered to express a CAR specific to CD19, a protein found on the surface of certain immune cells and cancer cells, show antigen‐specific activation, degranulation, cytotoxicity, and cytokine secretion comparable with that of conventional CD19‐CAR T‐cells and maintain homogeneous expression of TCRs derived from initial clones [106].

Importantly, organoids are increasingly used in CAR‐related evaluations. A recent assay tested CAR NK cell‐mediated cytotoxicity against cancer organoids [31]. The investigators engineered CAR NK‐92 cells targeting epidermal growth factor receptor variant III, a neoantigen expressed by solid cancers [108]. They established robust assays for continuous cell‐resolved analysis at the individual organoid level to monitor effector recruitment and cytotoxicity using luciferase‐based endpoint measurements or in vivo microscopy [31]. Another study demonstrated that CAR T‐cell‐derived tumor necrosis factor is a potent antitumor effector, synergistic with Smac mimetics [109]. CAR T‐cell therapy has also been evaluated in glioblastoma and bladder cancer organoids [20, 51, 110]. Glioblastoma organoids preserve the original TME, but their large volume hinders killing by CAR T‐cells because they are generated from tumor tissue fragments in culture medium [111]. In contrast, bladder cancer organoids are produced from single cells and are more efficiently destroyed by CAR T‐cells [110]. The blood‐brain barrier hinders the distribution of antitumor drugs and immune cells, particularly in patients with nonsmall cell lung cancer and brain metastases. Similarly, Li et al. [112] demonstrated that B7‐H3.CAR T‐cells exhibit antitumor activity against lung cancer organoids in vitro. Co‐expression of the CCR2B receptor significantly improves the ability of these cells to cross the blood‐brain barrier and enhances their antitumor activity against tumor lesions in the brain [112]. Therefore, PDTOs represent a promising preclinical approach for the evaluation of patient‐specific responses to CAR therapy.

#### 3.2.3 Extending the therapeutic reach of immunotherapy in organoids

In addition to ICIs and adoptive cell therapy, several new immunotherapy regimens have shown promise. These include immunomodulatory monoclonal antibodies, bispecific antibodies, and oncolytic virus therapy.

Co‐culture organoids can be used to evaluate the efficacy and mode of action of immunomodulatory monoclonal antibodies. For example, a co‐culture model using exogenous immune cells and CCR PDOs identified that the NKG2D‐MICA/B pathway was involved in the destruction of tumor cells [80]. After the anti‐MICA/B treatment, there was an increase in expression of the inhibitory receptor NKG2A expressed by CD8^+^ T and NK cells. Thus, the combination of anti‐MICA/B and anti‐NKG2A has a synergistic effect.

Bispecific antibodies, which can bind two different antigens simultaneously, can enhance recognition or bring cytotoxic cells and target tumors into proximity, resulting in a cytotoxic effect in a tumor [113]. One study assessed sensitivity to the bispecific antibody cibisatamab in multidrug‐resistant metastatic CRC PDOs co‐cultured with allogeneic CD8^+^ T‐cells [114]. Cibisatamab binds to carcinoembryonic antigen (CEA) on cancer cells and CD3 on T‐cells, triggering the destruction of cancer cells expressing moderate to high levels of CEA by T‐cells [114]. Live confocal microscopy was used to examine redirected killing in colon cancer, facilitating an ongoing clinical trial of cibisatamab [115]. Another study established co‐cultures of high‐grade serous ovarian cancer organoids with autologous immune cells treated with a bispecific anti‐PD‐1/PD‐L1 antibody. Transcriptional analysis suggested that the increased efficacy of immunotherapy in ovarian cancer is driven by changes of state in small subsets of NK and CD8^+^ T‐cells [116].

Oncolytic viruses are a newly identified class of immunotherapeutic agents with strong potential as immunostimulators [117, 118, 119, 120, 121]. One study showed that PDOs can be used to screen for sensitivity to oncolytic adenovirus alone or in combination with chemotherapy [122]. Another study found that the Zika virus has oncolytic activity in glioblastoma stem cells [123]. Using glioblastoma‐brain cortical organoids, Zhu et al. [124] demonstrated that the Zika virus preferentially infects and destroys glioblastoma stem cells in a SOX2‐dependent manner. Meanwhile, Hamdan et al. [125] designed an oncolytic adenovirus secreting a cross‐hybrid Fc fusion peptide targeting PD‐L1. When used in a co‐culture model of renal cell carcinoma PDOs and PBMCs, the oncolytic virus triggered the effector mechanisms of IgG and IgA1, activating neutrophils and resulting in increased tumor organoid destruction. In conclusion, PDTOs are a potentially valuable tool for the evaluation of the killing efficacy of oncolytic viruses in clinical settings and for studying relevant mechanisms [126].

### 3.3 Identification of biomarkers in organoids for prediction of a patient's response

Immunotherapy is not always effective [87]. Therefore, identifying biomarkers that predict the likelihood of a successful response to immunotherapy has been a focus of research [127]. The development of organoid models provides a new research tool for identifying such biomarkers (Figure 3).

Biomarkers of a response to therapy have been identified in the TME, including cellular components, functions, surface molecules, and location. For example, co‐culture of CRC PDTOs with autologous expanded TILs has been used as an individualized preconditioning test platform to predict a patient's response to neoadjuvant chemoradiotherapy [32]. Cytotoxicity in co‐cultures was significantly higher in patients with a complete response to chemoradiotherapy than in nonresponders. This finding allows for patient stratification and suggests that the function of TILs is a strong biomarker of the response to therapy [32]. Notably, Chen et al. [128] successfully cultured CRC ALI PDTOs, treated them with an anti‐PD‐1 agent and found that the level and activity of tumor‐infiltrating MDSCs were important markers of the efficacy of immunoblockade therapy. Similarly, using co‐cultures of PDAC PDOs and autologous immune cells, Holokai et al. [129] demonstrated that increased infiltration of polymorphonuclear cells and MDSCs in the TME suppresses T‐cell effector function regardless of PD‐1/PD‐L1 inhibition. Recently, PDTOs have been used to identify markers of effective antitumor immune activity. For example, HLA‐II expression in PDTOs was found to be associated with a good survival outcome[130]. K‐means clustering analysis based on HLA‐II expression in PDTOs identified a group of patients with intrinsic immunogenicity in cancer cells characterized by high expression of features associated with HLA‐I, HLA‐II, antigen presentation, and immunostimulation [130]. Furthermore, Voabil et al. [44] found that tumor immune composition in response to PD‐1 blockade could be predicted using five parameters on the PDTF platform, namely, CXCL13 levels, PD‐1 high‐expression B cells, and the number and size of tertiary lymphoid structures. These findings are consistent with those of previous studies and highlight the need to reevaluate our understanding of biomarkers of the outcome of PD‐1 blockade [22]. Indeed, researchers found that in HCC PDOs, both CAR T‐cells expressing CD39 and tumor‐reactive CD8^+^ T‐cells induced more tumor cell apoptosis than T‐cells without CD39 expression, indicating that CD39 may be a marker of T‐cell effectiveness [131]. Surprisingly, a study that analyzed patients' neoantigens (*n* = 56) using a personal cancer vaccine tool based on the MHC‐I algorithm and whole‐exome sequencing found that high‐affinity neoantigens (HANs), defined as mutant neoantigens with an IC_50_ < 50 nM, were positively correlated with better overall survival in patients with HCC [132]. HANs activate tumor‐reactive CD39^+^ CD8^+^ T‐cells, which trigger antitumor activity, and patients in the HAN‐high group benefited more from anti‐PD‐1 therapy than those in the HAN‐low group [132].

### 3.4 Drug screening in organoids

Traditional clinical regimens, including chemotherapy, immunotherapy, and targeted drugs, are not effective in all patients because of individual differences. Personalized precision therapy requires preclinical drug screening[133, 134]. Co‐culture models have been widely used for preclinical testing of medications (Figure 3). For example, the effectiveness of customized immunotherapy for appendiceal cancer has been assessed in preclinical trials using an organoid platform [135]. Unsorted tumor cells were used to create PDTOs, with or without the addition of patient‐matched immune components, to create immune cell‐enriched PDTOs. After being cultured for 7 days, PDTOs were treated with immunotherapy, after which their effectiveness was evaluated. Notably, according to the study, there was a significant decrease in the viability of high‐grade appendiceal organoid cells in response to treatment with pembrolizumab [135]. Similarly, a recent study revealed that agents from the apoptosis inhibitor and histone deacetylase inhibitor classes identified by drug library screening upregulated MHC‐I neuroblastoma, enhancing its immunogenicity driven by T‐cells and NK cells [136]. Furthermore, there has been a study in which co‐culture models were used to screen for small‐molecule compounds that enhance the immune response [137]. The study found that atractylenolide I significantly promoted tumor antigen presentation in human and mouse CRC cells, enhancing cytotoxicity in CD8^+^ T‐cells [137]. Interestingly, Dong et al. [138] developed PDTOs by encapsulating multicellular liver tumor clusters in hydrogel capsules, performed personalized preclinical drug screening and observed interindividual differences in the sensitivity of the PDTOs.

In conclusion, organoid models have the features of simplicity, affordability, success, speed, and high throughput, which position them as a platform capable of driving personalized drug screening, refining clinical precision, and improving cost‐effectiveness, operational efficiency, and progression of precision medicine.

### 3.5 Infection models in organoids

Chronic infection and inflammation can promote tumor progression and resistance to treatment [139]. To study this process, researchers have established organoid infection models for various diseases (Figure 3). For example, liver organoids have been used to model hepatitis B and C virus infections [140, 141, 142]. Baktash et al. [142] imaged the entry of the hepatitis C virus into a 3D polarized hepatoma system and demonstrated that the virus engages entry factors through actin‐dependent mechanisms. Similarly, functional liver organoids generated from human‐induced PSCs [140] and healthy donor liver tissues [141] have been used as models of hepatitis B virus infection.

Bacterial infections have also been studied using organoid models. Pleguezuelos‐Manzano et al. [143] exposed human intestinal organoids to genotoxic *Escherichia coli* by repeated luminal injection over 5 months and found that prolonged exposure resulted in a unique signature of mutations, suggesting that detecting and removing polyketide synthetase‐positive *E. coli* and re‐evaluating probiotic strains containing polyketide synthetase islands could reduce the risk of cancer in a large population. Likewise, research has shown that the gastric epithelium actively recruits human monocyte‐derived DCs for immunosurveillance through chemokine‐dependent mechanisms, with increased recruitment during active *Helicobacter pylori* infection [81], which is a major risk factor for gastric cancer [144]. Subsequently, a mouse or human Transwell co‐culture system revealed a role for the Toll‐like receptor 9 pathway in mediating *H*. *pylori* ‐induced gastric chemotaxis [145]. Holokai et al. [82] developed gastric cancer PDOs grown in Matrigel with DCs and CTLs purified from PBMCs to study PD‐L1/PD‐1 interactions between the gastric epithelium and the host's immune response during *H*. *pylori* infection. They found that inhibiting PD‐L1/PD‐1 interaction induced the proliferation of CTLs and the destruction of organoids. However, not all microorganisms cause tumors. For example, Gao et al. [146] found that increased levels of *Fusobacterium nucleatum* in PDTOs were associated with an improved therapeutic response to PD‐L1 blockade. In summary, organoids have emerged as a promising model for studying the effects of viral or bacterial infections on the development and progression of tumors, as well as for evaluation of the efficacy of potential treatments for these infections.

## 4 LIMITATIONS AND PERSPECTIVES

PDTOs are deepening our understanding of the heterogeneity of cancer and its implications for personalized medicine because of their ability to retain the genetic, proteomic, morphological, and pharmacological characteristics of the parent tumor while allowing unprecedented genomic and environmental manipulation [147]. Immunotherapy research drives the development of novel cancer therapies by targeting immune regulatory pathways [86]. However, the main disadvantage of organoid technology when used to predict immunotherapy is the absence of stromal compartments, blood vessels, and immune cells [28]. To overcome this limitation, more complex organoid co‐cultures that include immune cells [39, 135], CAFs [70, 71], and blood vessels [83] have been developed. Furthermore, comprehensive protocols have been established to maintain the original TME, encompassing PDTFs, ALI, suspension 3D culture, and ToC models. These co‐cultures have potential in terms of mimicking the efficiency and resistance mechanisms involved in immunotherapy (Table 1). There are also other limitations of organoid co‐culture models to consider (Table 2).

**Table 1:** Co‐culture of organoids for different research purposes by different research groups.

Cancer type	Cell type	Purpose	Filter or culture type	Readout	Material of ECM	References
Lung cancer	PBMCs and PDTOs from NSCLC	To obtain tumor‐reactive T cells by co‐culture of PBLs with matched PDTOs	100 μm	Flow cytometry for evaluation of MHC‐I and PD‐L1 expression by PDTOs and quantification of organoid‐induced IFNγ production and CD107a cell surface expression of CD8^+^ T cells	Geltrex	[21]
Lung cancer	PDTOs and TILs of NSCLC patients	To combine immunotherapy with MEK‐I	S1: >100 μm; S2: 30–100 μm; S3: <30 μm	Western blot analysis of protein; real‐time qPCR analysis of IFNγ, IL‐12, IL‐10, IL‐1β, IL‐6, and TNF‐α; MTT cell proliferation assays	Matrigel	[92]
Bladder cancer	PDTOs of bladder cancer with CAR‐T cells from PBMCs	To establish an in vitro technological platform to evaluate CAR‐T cell‐mediated cytotoxicity against bladder cancer	70 μm	Immunohistochemistry for CAR‐recognizable targets; cytotoxic assays and ELISA to test the efficacy of CAR‐T cells	50% Matrigel	[111]
Chordoma	Cancer cells and TILs of human chordoma specimen	To be a potential model to predict response to PD‐1/PD‐L1 checkpoint inhibitors	−	Assessment of diameters, PD‐L1 expression, and percentages of DAPI‐stained cells of PDTOs	2% matrigel	[91]
Cholangiocarcinoma	Cancer cells and TILs from fresh tumor tissue samples and PBMCs	To develop a co‐culture method with CCA PDTOs and immune cells to represent anticancer immunity in vitro	−	Flow cytometric, time‐lapse confocal analysis, and CYFRA quantification assay to quantify cell death	BME	[34]
Hepatocellular carcinoma	TILs and HCC PDTOs from needle biopsy and APCs from PBMCs	To define the function of TMB or neoantigens in antitumor immunotherapy	70 µM	Microphotograph images and flow cytometry to analyze killing efficiency by FITC signal (caspase 3/7 probe), CD107a, and CD137 on CD39^+/^^−^ CD8^+^ T cells; IFN‐γ production was determined by ELISPOT assay	BME	[133]
Glioblastoma	GBOs from resected patient glioblastoma tissue and CAR T cells	To establish methods for generating and biobanking GBOs that recapitulate the key features of their corresponding parental tumors and model CAR‐T cell immunotherapy	Tumor pieces in medium and were placed on an orbital shaker rotating at 120 rpm	Immunostaining and quantifications of averaged signal intensity of CD3, cleaved‐caspase‐3 (CC3), granzyme B, and averaged EGFRvIII/EGFR signal intensity ratio in GBOs after co‐culture; ELISA for cytokines interleukin (IL)‐2, TNF‐α, and interferon (IFN)‐γ revealed	50% DMEM: F12 and 50% Neurobasal	[20, 52]
Pancreatic ductal adenocarcinoma	Human MDSCs, DCs, and CTLs of PBMC with PDOs	To develop a model to specifically target mechanisms that deplete MDSCs as a therapeutic strategy for PDAC	70 µM	Percent of proliferating CTLs, and CD8^+^perforin^+^‐expressing cells	Matrigel	[130]
Pancreatic ductal adenocarcinoma	Murine and human PSCs and PDAC PDTOs	To provide direct evidence for CAF heterogeneity in PDAC biology	−	Secretion of inflammatory cytokines, RNA ISH, RNA sequencing analysis	Matrigel	[71]
Pancreatic cancer	PBMCs, CAFs, and cancer cells of human pancreatic cancer	To develop and characterize PDTOs and multi‐cell type organotypic co‐culture models	−	Immunofluorescence, organoid histology	Matrigel	[80]
Pancreatic ductal adenocarcinoma	Healthy pancreas and PDAC PDTOs	To explore the feasibility of using PDTOs as a screening platform for the oncolytic adenovirus (OA) response	−	Colorimetric MTT assay for cytotoxicity assays, organoid karyotyping	Matrigel	[123]
Ovarian cancer	PDTOs from human high‐grade serous ovarian cancer (HGSC)	To study the mechanism of action of the ICIs	−	scRNA‐seq analysis of all immune cell types post‐ICB treatment	Matrigel	[117]
Gastric cancer	GC PDTOs and PBMCs	To verify that dexamethasone can enhance the efficacy of ICIs	−	Expression levels of CC3 and CC8	Matrigel	[97]
Gastric cancer	Human‐derived gastric epithelium, DCs, and luminal *Helicobacter pylori* bacteria	To address how antigen‐presenting cells are recruited to and interact with the gastric epithelium to access *H. pylori* antigens	−	Particle tracking analysis of time‐lapse images; transwell chemotaxis assays	Matrigel	[82]
Gastric cancer	*H. pylori*, PDTOs, and autologous immune cell	To investigate the mechanism that how *H. pylori* induce PD‐L1 expression on gastric epithelial cells	−	Immunofluorescence, western blots, qRT‐PCR, flow cytometry	Matrigel	[83]
Colorectal cancer	Tumor specimens derived from CRC or lung cancer	To investigate and characterize resected tumor tissue in the presence of stromal and immune cells	ALI	IHC for CA19‐9, CEA, CD45 and CD3; assessment of CEA and CA19‐9 in 3D culture media; CellTiter‐Glo (ATP content) was used to determine the dose‐response characteristics	Matrigel	[23]
Colorectal cancer	MDSCs isolated from healthy cord blood, bulk T cells purified from the PBMCs of healthy donors, and primary tumor tissues	To investigate whether and how tumor‐infiltrating MDSCs are shaped in response to anti‐PD‐1 treatment and what their impact on therapeutic efficacy is in CRC	ALI (primary tumor tissues were minced into 125–500 mm^3^ fragments)	The expression of Annexin V and DR5 on MDSCs was determined using fluorescence‐activated cell sorting (FACS) in CRC tissues; apoptosis‐related receptors were examined in MDSCs using qPCR	Type I collagen gel	[129]
Colorectal cancer	TILs and tumor cells derived from patients with CRC	To assess the infiltration, activation, and function of T and NK cells toward human colorectal tumors	The adherent cells were isolated and seeded in ultra‐low attachment 96 wells plates	Tumor volume, shape, and infiltration by immune cells; precise flow cytometry phenotyping of T and NK cells infiltrating human tumor spheroids	No	[81]
Colorectal cancer	CAR‐engineered NK‐92 cells, EGFRvIII‐CAR NK‐92 cells, normal human colon pathological mucosa, and primary CRC tumor tissues	To establish a quantitative platform for CAR‐mediated cytotoxicity toward patient‐derived colon organoids	−	Luciferase activity as a quantitative read‐out for lysis‐resistant target cells; live imaging strategy using spinning‐disk microscopy; replating assay to analyze the presence of viable tumor cells	Matrigel	[31]
Colorectal cancer	PBMCs and tumor tissues from dMMR CRC	To obtain tumor‐reactive T cells by co‐culture of PBL with matched tumor organoids; whether such T cells can be used to assess the efficiency of tumor cell killing	100 μm	Flow cytometry for evaluation of MHC‐I and PD‐L1 expression by tumor organoids and quantification of organoid‐induced IFNγ production and CD107a cell surface expression of CD8^+^ T cells	Geltrex	[21]
Colorectal cancer	Multidrug‐resistant metastatic CRCs and allogeneic CD8 T cells from PBMCs	To enable more detailed insights into mechanisms of cibisatamab resistance and sensitivity	70 μm	Surface CEA expression analysis by flow cytometry, cancer cell growth assessment by immunofluorescence microscopy, gene expression analysis	Matrigel	[115]
Rectal cancer	TILs and cancer cells from tumor tissue of rectal cancer	To test TIL cytotoxicity in patients and demonstrate the rescue of TIL function after checkpoint inhibition blockade	−	Measuring the mean fluorescence intensity of the cell death marker, propidium iodide to evaluate TIL‐mediated tumoroid lysis	Matrigel	[32]
Breast cancer	VeraVec HUVEC‐TURBO‐GFP cells, primary human breast tumor specimens	To assess tumor–vessel interactions and understand the mechanisms by which mosaic vessels form	Microvessel model	Measurement of permeability and focal leaks; endothelial cell proliferation and cell death analysis	Collagen‐I gel	[84]
Melanoma	Fresh tumor specimens	To develop a platform to evaluate tumor‐immune interactions in 3D microfluidic culture	S1: >100 μm; S2: 40–100 μm; S3: <40 μm Microfluidic 3D culture	Flow cytometry, serial microscopy (live/dead analysis), bioplex cytokine profiling of conditioned media	Collagen hydrogels	[59]
Melanoma	Melanoma and lymph node biospecimens from the same patient and patient‐matched T cells from PBMCs	To verify the hypothesis that engineering a combined lymph node/melanoma organoid from the same patient would allow it to remain viable for personalized immunotherapy screening	100 μm	Live/dead staining and quantitative metabolism assays recorded relative drug efficacy	ECM‐mimicking HA/collagen‐based hydrogel	[40]
Renal cell carcinoma	Renal cell carcinoma tissue samples and PBMCs	To evaluate the efficacy of the novel oncolytic adenovirus expressing enhanced cross‐hybrid IgGA Fc PD‐L1 inhibitor	−	Using calcein green for visualizing cell viability, FACS analysis of PD‐L1 expression, and LDH release assays for measuring cell killing	30% Matrigel	[126]

Abbreviations: PBMCs, peripheral blood mononuclear cells; PDTOs, patient‐derived tumor organoids; TILs, tumor‐infiltrating lymphocytes.

**Table 2:** The limitations of current organoid co‐culture research.

Source	Existing problems	Existential challenges	Possible strategies	References
Biological samples	The success rate of organoid culture is unstable	Tumor type and starting material	Explore ideal culture conditions and techniques tailored to diverse tumor types; streamline the handling and preparation of initial materials	[148]
	PDTOs cannot capture broad patient‐specific cancer biological heterogeneity	Single biopsies or small fragments of surgically resected tissue	Select representative tissue samples from different tumor regions; employ various collection methods	[148]
Culture strateges	Current culture technology is limited	Expensive growth factors and conditioned media	Seek economical and efficient alternatives; develope culture mediums devoid of animal‐derived components; investigate tissue engineering and cell engineering techniques	[149]
	Nonstandardized and ill‐defined culture protocols	The animal‐derived scaffolds mostly used are unclear and poorly tunable; the animal‐derived serum introduces xenogeneic components	Explore artificially synthesized scaffolds	[149]
TME	The culture selection affects the growth of tumor clones	PDTOs are not exposed to external stresses that occur in situ, such as hypoxia or immune selection	Use a low‐oxygen culture chamber or introduce immune cells and cytokines; transplant in vitro‐cultured PDTOs into animal models	[44]
	Robust drug screening and testing require the reproducible quantitative studies	Biophysical cues in TME have a profound effect on cell and tissue physiology, as well as important factors leading to immunotherapy resistance	Control the size, shape, and relative arrangement of different cell types within organoids and biophysical factors	[150, 151, 152]
	Need to better mimic the cell components, structural and physical characteristics of the TME	The stromal and immune components cannot be sustained for an extended period	Explore using a wider variety or combinations of cytokines; explore more complex or multilayered three‐dimensional scaffolds or matrices	[153, 154]

Abbreviations: PDTOs, patient‐derived tumor organoids; TME, tumor microenvironment.

First, the success rate of organoid culture varies according to tumor type and starting material [155]. The establishment of organoids in limited quantities presents a challenge in terms of their application in high‐throughput drug screening. Addressing these problems necessitate an exploration of ideal culture conditions and techniques tailored to diverse tumor types, as well as streamlining the handling and preparation of initial materials to ensure their integrity and purity. Employing these approaches can enhance the success rate of organoid culture across various tumor types and starting materials while preserving their biological attributes and heterogeneity [148].

Second, capturing the patient‐specific heterogeneity of cancer biology using organoids requires the sourcing of tissue samples that reflect the spatiotemporal diversity of tumors. Hence, researchers need to optimize their sample collection strategies to encompass the spatiotemporal diversity of tumors. This involves selecting representative tissue samples from different tumor regions and using various collection methods, such as surgical specimens and biopsies. Establishing a suitable sample repository for research purposes is also essential. Using these approaches, it becomes possible to better capture the patient‐specific biological diversity of tumors, providing more valuable sample resources for tissue cultivation studies.

Third, there are presently several technological limitations when using organoid models, including the expense of growth factors and conditioned medium and the fact that the use of animal‐derived serum introduces xenogeneic components [156]. Researchers could explore various strategies that could enhance the feasibility and reliability of their experiments and address these challenges. Such approaches include seeking more economical and efficient alternatives to costly growth factors, developing a culture medium devoid of animal‐derived components, replacing expensive growth factors with cytokines and small molecules, investigating tissue engineering and cell engineering techniques to reduce dependency, and adopting serum‐free medium or human‐sourced serum. Implementing these measures could help to overcome the current limitations related to cost and heterogeneity, thereby improving the practicality and reliability of experimental outcomes.

Fourth, most 3D in vitro cancer organoid experiments rely on animal‐derived scaffolds that are unclear and poorly tunable [156]. To tackle this issue, the adoption of artificially synthesized scaffolds instead of animal‐derived scaffolds could be explored to enhance the clarity and adjustability of 3D tumor‐like organoid experiments [149, 157]. These synthetically engineered scaffolds can be tailored and optimized according to the type of tumor and microenvironmental factors, better mimicking the in vivo state and behavior of tumors. Furthermore, the use of synthetic scaffolds may mitigate potential concerns associated with heterogeneity, immune reactions, and contamination that can arise from animal‐derived scaffolds [153, 158].

Fifth, PDTOs are not exposed to external stresses that occur in situ, such as hypoxia or immune selection [43]. One solution involves cultivating PDTOs in vitro using a low‐oxygen culture chamber or introducing immune cells and cytokines to create an ex vivo system that closely resembles the in vivo environment, thereby simulating hypoxia or immune selection conditions. Another approach entails transplanting in vitro‐cultured PDTOs into animal models, allowing them to be re‐exposed to in vivo hypoxic or immune selection pressures.

Sixth, biophysical cues in TME have recently been recognized as important features of cancer. Biophysical factors such as structure, stiffness of the ECM, tumor interstitial fluid pressure, solid stress, and vascular shear stress can profoundly affect cell and tissue physiology [150, 159] and lead to resistance to immunotherapy [151]. Therefore, controlling the size, shape, and relative arrangement of different cell types within organoids and biophysical factors is important to allow the reproducible quantitative studies required for robust drug screening and testing.

Seventh, even with the addition of IL‐2, anti‐CD3, and anti‐CD28 antibodies, the stromal and immune components in a co‐culture model of tumor organoids cannot be sustained for an extended period. To address this issue, we can use a wider variety of combinations of cytokines, such as IL‐15, IL‐21, and IFN‐γ, to enhance the proliferation and efficacy of immune cells. On the other hand, more complex or multilayered 3D scaffolds or matrices could be employed, such as collagen, glass fibers, and polylactic acid, to better mimic the structural and physical characteristics of the TME [152, 158]. Importantly, when using organoid co‐culture models, combination therapy that includes immunotherapy, targeted agents, and chemotherapy drugs is sometimes used to enhance efficacy but the toxicity of chemotherapy is not assessed. It is important to bear in mind that the outcomes of many clinical trials have been determined by the efficacy and safety profiles of chemotherapeutic agents. Therefore, while using organoid co‐culture models to study the efficacy of combination therapy, the toxicity of chemotherapy is also worth evaluating.

When these problems are solved, organoid‐based research methods can be expected to promote basic science and translational research in immuno‐oncology. Moreover, the use of organoid imaging techniques to study the dynamics of the interaction between tumor cells and inflammatory cells is promising [154]. PDTO will pave the way to realizing the promise of human tumor immunotherapy and personalized treatment.

## AUTHOR CONTRIBUTIONS

**Zhaoru Gu**: Writing—original draft (equal); writing—review and editing (equal). **Quanyou Wu**: Visualization (equal). **Bingqing Shang**: Visualization (equal). **Kaitai Zhang**: Funding acquisition (equal); visualization (equal). **Wen Zhang**: Conceptualization (equal); funding acquisition (equal); resources (equal); supervision (equal); visualization (equal); writing—review and editing (equal).

## CONFLICT OF INTEREST STATEMENT

The authors declare no conflict of interest.

## ETHICS STATEMENT

The authors have nothing to report.

## INFORMED CONSENT

The authors have nothing to report.

## Data Availability

Data sharing not applicable to this article as no data sets were generated or analysed during the current study.
